# Supplementary material for: Viral Transcript and Tumor Immune Microenvironment-Based Transcriptomic Profiling of HPV-Associated Head and Neck Squamous Cell Carcinoma Identifies Subtypes Associated with Prognosis
Source: Viruses. 2024 Dec 24;17(1):4. doi: 10.3390/v17010004 (PMC11769425; doi:10.3390/v17010004)

Figure S1.Validation of TME subtypes

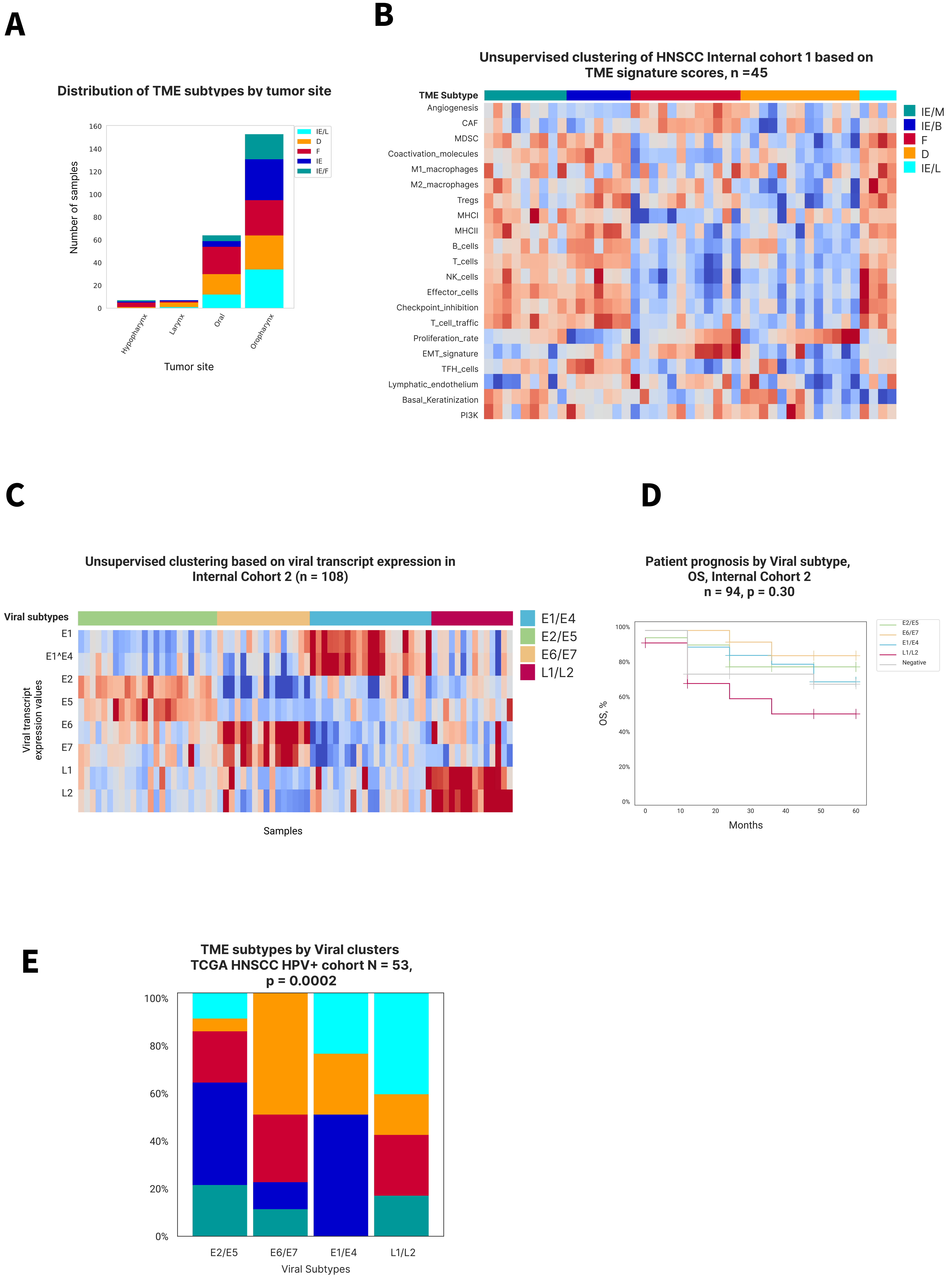

Figure S2. Differential genomic events in HPV+ vs HPV- HNSCCs.

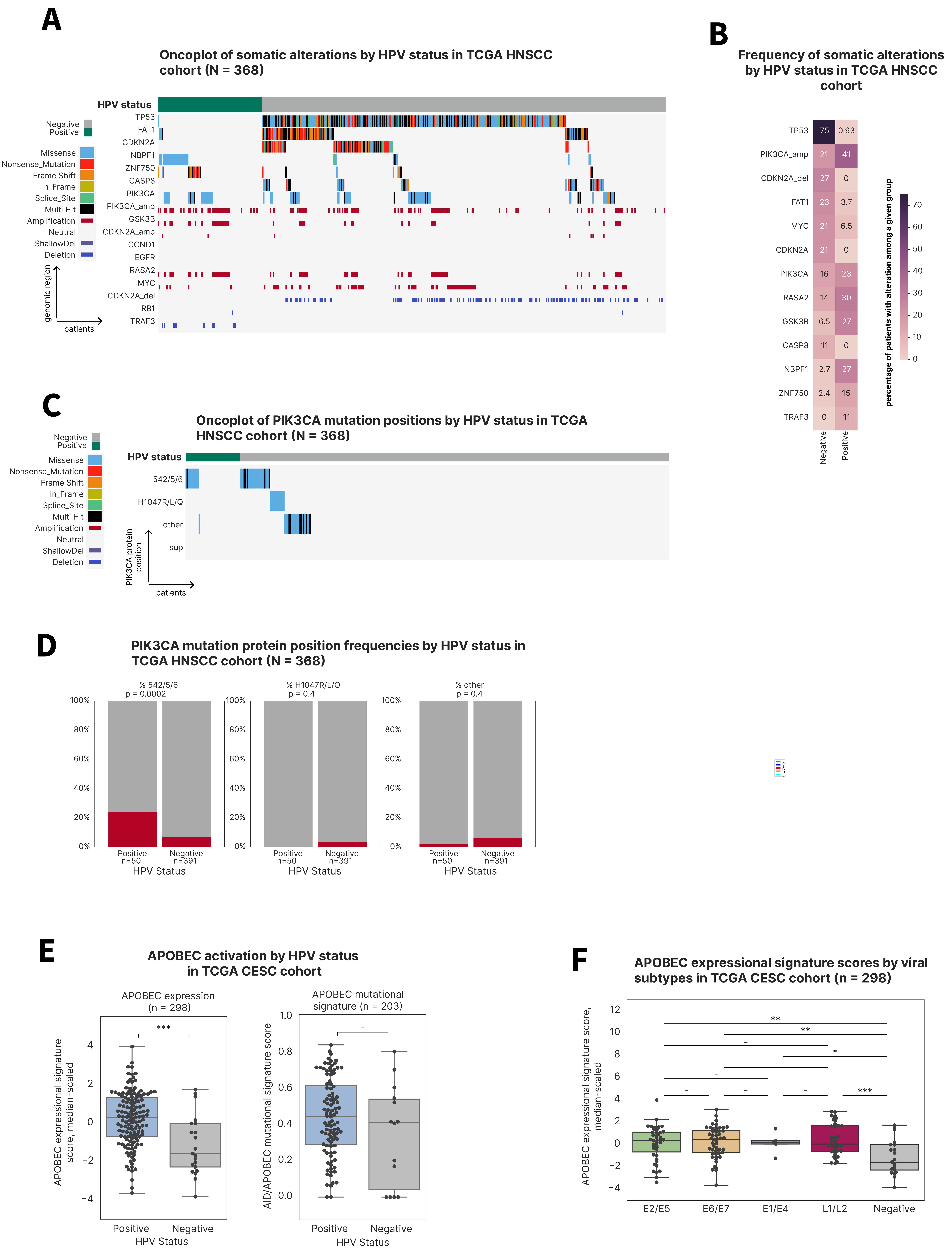

Supplement: Supplementary file 1 [file viruses-17-00004-s001.zip › Supplemental figures.pdf]
